# Multitargeting the Action of 5-HT_6_ Serotonin Receptor Ligands by Additional Modulation of Kinases in the Search for a New Therapy for Alzheimer’s Disease: Can It Work from a Molecular Point of View?

**DOI:** 10.3390/ijms23158768

**Published:** 2022-08-07

**Authors:** Kinga Czarnota-Łydka, Katarzyna Kucwaj-Brysz, Patryk Pyka, Wawrzyniec Haberek, Sabina Podlewska, Jadwiga Handzlik

**Affiliations:** 1 Department of Technology and Biotechnology of Drugs, Medical College, Jagiellonian University, Medyczna 9, 30-688 Krakow, Poland; 2 Doctoral School of Medical and Health Sciences, Jagiellonian University Medical College, Św. Łazarza 15, 31-530 Krakow, Poland; 3 Maj Institute of Pharmacology, Polish Academy of Sciences, Department of Medicinal Chemistry, Smętna Street 12, 31-343 Krakow, Poland

**Keywords:** 5-HT_6_ ligands, Alzheimer’s disease, dementia, MARK4, ROCKI, ROCKII, CDK5

## Abstract

In view of the unsatisfactory treatment of cognitive disorders, in particular Alzheimer’s disease (AD), the aim of this review was to perform a computer-aided analysis of the state of the art that will help in the search for innovative polypharmacology-based therapeutic approaches to fight against AD. Apart from 20-year unrenewed cholinesterase- or NMDA-based AD therapy, the hope of effectively treating Alzheimer’s disease has been placed on serotonin 5-HT_6_ receptor (5-HT_6_R), due to its proven, both for agonists and antagonists, beneficial procognitive effects in animal models; however, research into this treatment has so far not been successfully translated to human patients. Recent lines of evidence strongly emphasize the role of kinases, in particular microtubule affinity-regulating kinase 4 (MARK4), Rho-associated coiled-coil-containing protein kinase I/II (ROCKI/II) and cyclin-dependent kinase 5 (CDK5) in the etiology of AD, pointing to the therapeutic potential of their inhibitors not only against the symptoms, but also the causes of this disease. Thus, finding a drug that acts simultaneously on both 5-HT_6_R and one of those kinases will provide a potential breakthrough in AD treatment. The pharmacophore- and docking-based comprehensive literature analysis performed herein serves to answer the question of whether the design of these kind of dual agents is possible, and the conclusions turned out to be highly promising.

## 1. Introduction

Reduced cognitive capacity is a growing problem, becoming as common as diseases such as obesity, cancer and heart disease. Cognitive decline usually, but not always, occurs in elderly people and may have light symptoms such as forgetting information and difficulty in memorizing situations; these light symptoms are categorized as mild cognitive impairment (MCI) [1,2]. Although such symptoms are not extremely problematic in everyday life, they may be the first sign of developing dementia, a serious cognitive dysfunction leading to obstacles in performing simple tasks and very often to loss of functional independence [3]. Worryingly, about 50 million people in the world suffer from dementia [4]. Interestingly, 70% of reported cases are a consequence of Alzheimer’s Disease (AD), i.e., the complex central nervous system disorder, occurring usually after 60 years of age [5]. Unfortunately, despite the huge number of studies, the available small molecule drugs used for treatment have significant limitations: improvement in cognition is observed for only a short time and the drugs treat only the symptoms of the disease, rather than its influence or causes [6]. Their pharmacodynamic action concentrates on two protein targets: (i) inhibition of acetylcholinesterase (AChE) in the case of donepezil, galantamine and rivastigmine, and (ii) antagonism towards N-methyl-D-aspartate receptor (NMDA) for memantine [7]. In 2021, after almost a twenty-year break, the FDA approved the first disease-modifying option for AD treatment, the monoclonal antibody aducanumab, which gave hope for a breakthrough in AD therapy [8]. However, the FDA’s accelerated acceptance of this pathway caused a significant amount of controversy in the scientific community, as the results from different clinical studies were not consistent, thus not clearly confirming the therapeutic efficacy [9]. Moreover, the approval was mainly based on the ability of aducanumab to remove β-amyloid (Aβ) plague, whereas it has not yet been proved that Aβ clearance is correlated with cognition impairment. Finally, the cost of the treatment and the safety profile were also problematic issues [10]. In this light, searching for novel small chemical entities acting via non-standard protein targets is highly urgent to deal with this still unmet clinical need.

On the other hand, in the last few decades, serotonin 5-HT_6_ receptor (5-HT_6_R) and its ligands have been strongly investigated in terms of potency to fight cognition dysfunctions [11,12]. In many preclinical studies, it has been confirmed that both agonism and antagonism of 5-HT_6_R influences improvement in cognition capacity [13]. However, all the selective 5-HT_6_R agents that reached clinical studies were sub-efficacious in AD patients [14]. This disappointing lack of translatability of preclinical findings to human applications has encouraged the development of novel therapeutic strategies. One of them concerns polypharmacology, i.e., an approach that, opposite to the paradigm “one drug, one target”, aims at designing molecules with action via two or more therapeutic proteins. It is supposed that the clinical failure of 5-HT_6_R agents is a consequence of the fact that those anti-AD drug candidates have been designed as single-target molecules, whereas such a complex disease as AD involves many different signaling pathways that form the pathological network [15]. Hence, selecting just one target may indeed lead to insufficiency [16]. Moreover, multidirectional molecules may have a more predictable pharmacokinetic profile than combined therapy with two or more single-target drugs [17,18]. These arguments have encouraged medicinal chemists to design and synthesize compounds with such a multifunctional profile. Based on the great potency in preclinical studies of the 5-HT_6_R ligands, it seems to be reasonable to combine such activity with targeting protein(s) that is/are also involved in the pathology of cognition processes. Until now, most of the reported multifunctional 5-HT_6_R ligands also have potency to inhibit the activity of cholinesterases (AChE—acetylcholinesterase and BuChE—butylcholinesterase [19]), dopamine D_3_ receptor [20] and other serotoninergic receptors, e.g., 5-HT_4_R [21].

Among the hallmarks of AD, the formation of the three following components: (i) the neurofibrillary tangles; (ii) the amyloid plaques and (iii) the insoluble Aβ, are the most important ones. Aβ is produced via the amyloidogenic degradation of the amyloid precursor protein (APP) with the contribution of both β and γ-secretases. Although the exact causes of AD are still unknown, the number of factors recognized as contributing to AD etiology increases every year and are assigned to various signaling pathways with specific kinases as the main players. Hence, this class of kinases should also be taken into consideration as therapeutic targets in the search for multifunctional anti-AD agents.

Given the urgent need for the development of innovative and effective procognitive therapies, this review is focused on computer-aided analysis of the possibility of designing novel multidirectional molecules with combined action via serotonin 5-HT_6_R and carefully selected kinases. Thus, (i) microtubule affinity-regulating kinase 4 (MARK4), (ii) Rho-associated coiled-coil-containing protein kinase I/II (ROCKI/II) and (iii) cyclin-dependent kinase 5 (CDK5) were considered for discussion, since these proteins are innovative as AD therapeutic targets, according to recent state of the art research, and, simultaneously, have not yet been considered in terms of combination with 5-HT_6_R activity.

## 2. 5-HT_6_R Ligands—Pharmacophore Features

### 2.1. Chemical Variety of 5-HT_6_R Ligands

**Figure 1:**
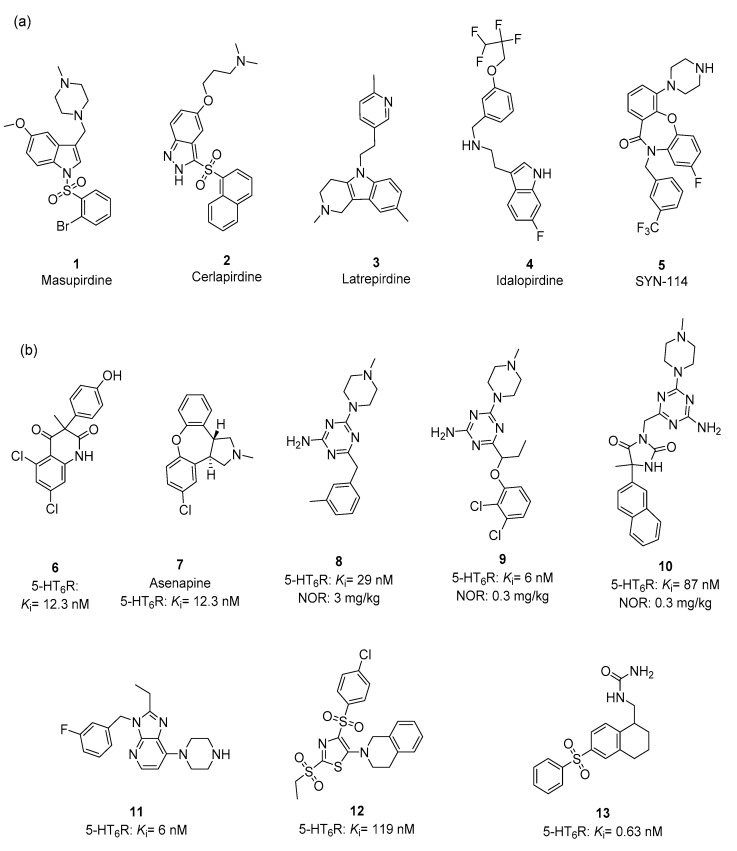
The structural variety of 5-HT_6_R antagonists: (**a**) compounds **1** [23,24], **2** [25], **3** [26,27,28,29], **4** [30,31,32] and **5** [33] investigated in clinical trials; (**b**) compounds in the early stages of R&D: the non-indole and non-sulfone derivatives **6** [34], **7** [35], **8** [36,37], **9** [38,39], **10** [40,41]and **11** [42] and non-basic antagonists **12** [43] and **13** [43,44]. The affinity for 5-HT_6_R expressed with *K*_i_ (nM). Procognitive effects in the Novel Object Recognition (NOR) test for **8–10**. at the dose shown [36,37,38,39,40,41].

Since the discovery of 5-HT_6_R in the mid-1990s, over a thousand of its ligands with the nanomolar affinity range and a diverse functional activity profile have been identified. Although this intrinsic activity does not define clear preferences for potential therapeutic actions, as procognitive, antidepressant-like and anxiolytic effects in animal models were confirmed either for agonists or antagonists, the 5-HT_6_R antagonists are of greater interest to researchers; 17 have reached clinical trials, including 6 towards AD [22]. On the other hand, the chemical space of 5-HT_6_R ligands is rather narrow, predominantly mapped by sulfonamide, indole and indole-like moieties, also containing a basic center most often represented by N-unsubstituted- or N-methylpiperazine (Figure 1).

According to the statistics provided in 2014, 80% of 5-HT_6_R ligands contain sulfone moieties, while 40% contain indole ones [45]. Thus, one of the two most advanced in clinical trials, masupirdine, consists of three aforementioned moieties (**1**) [23,24], while cerlapirdine (**2**) [25]is the sulfonamide derivative, two non-sulfone but indole-derived 5-HT_6_R antagonists, latrepirdine (**3**) [26,27,28,29] and idalopirdine (**4**) [30,31,32] failed in Phase III of clinical trials, and only one non-sulfone and non-indole derivative SYN-114 (**5**) [33] was active in Phase I (Figure 1a). Recent research results have contributed to increasing the chemical diversity of 5-HT_6_R ligands. Apart from the structures of quinoline-2,4-diones (**6**) [34] and asenapine (**7**) [35] described in 2008–2009, the last five years have also provided non-indole and non-sulfonic derivatives of triazine (**8**, **9**) [36,37,38,39], hydantoin (**10**) [40,41], imidazopyridine (**11**) [42], as well as non-basic 5-HT_6_R ligands (**12**, **13**) [43,44], which also showed procognitive activity in animal models (**8**–**10**), promising for potential AD therapies (Figure 1b).

Both ligand-based (LBDD) and structure-based drug design (SBDD) approaches are useful in the rational design of 5-HT_6_R ligands. However, a limitation to SBDD was the lack of a crystallographic structure for this receptor in PDB until now, thus condemning designs based on homology models. Last-minute lines of evidence [46] point to obtaining the first 5-HT_6_R crystal, which gives hope for increasing SBDD efficiency as soon as the crystallographic structure becomes available. Nevertheless, the ligand-based design has dominated the exploration of the chemical space for new 5-HT_6_R ligands so far, in which the pharmacophore model for antagonists developed by the team of Lopez-Rodriguez in 2005 seems to be the number one for the computer-aided LBDD, until now. 

### 2.2. Molecular Modeling Approaches to Evaluate the Potential 5-HT_6_R Compound Activity

We decided to combine both LBDD and SBDD to assess the potential of considered kinases inhibitors that also present 5-HT_6_R activity (detailed results are presented in the respective subchapters). At first, we used the pharmacophore-based method in order to examine the possibility of dual modulation of the 5-HT_6_R and each kinase selected from the considered ones (MARK4, ROCK I, ROCK II, CDK5) in a wider group of chemical compounds.

Taking into account the significant increase in the number of new highly active 5-HT_6_R antagonists for more than 15 years since the pharmacophore of Lopez-Rodriguez occurred, an update based on the extensive library of current antagonists (ligands) available in the CHEMBL database for the 5-HT_6_R pharmacophore model was made in the first step.

The developed pharmacophore was further used to analyze common structural features for the active kinase inhibitors and 5-HT_6_R.

For all 5-HT_6_R ligands with affinity to the receptor below 500 nM (expressed in *K_i_*, data fetched from the ChEMBL database) [47], the clustering procedure was carried out (with compounds represented by MOLPRINT2D fingerprint [48] and Tanimoto similarity metric used to measure distance between the clusters formed). The number of clusters was forced to be 50, and centroids together with compounds with the shortest distance to the centroid formed the set of compounds, which was used to construct the pharmacophore model (the total number of compounds was equal to 136). The clustering procedure was applied to ensure that the chemical space of ligands used for the pharmacophore model construction was representative to the whole set of 5-HT_6_R ligands to the highest possible extent.

**Figure 2:**
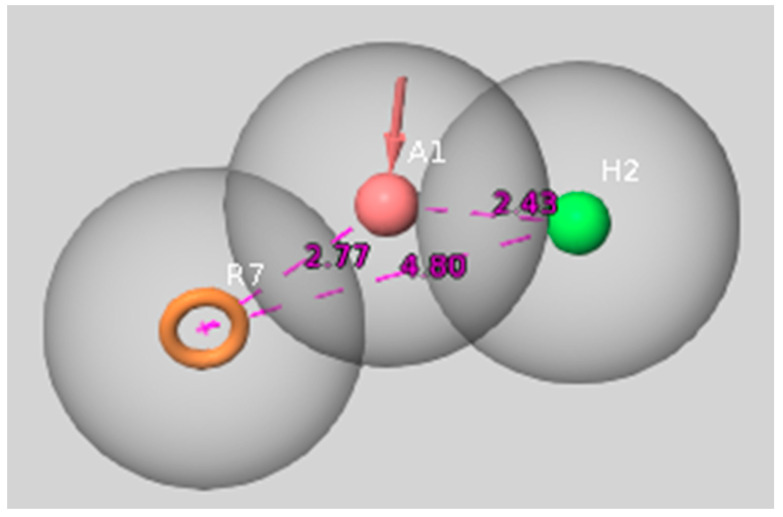
Pharmacophore model of the 5-HT_6_R ligands: aromatic moiety (R7), hydrogen bond acceptor (A1), hydrophobic moiety (H2).

The pharmacophore model was constructed using Phase [49] from the Schrödinger Suite 2022 (Figure 2).

Due to the relatively high fraction of 5-HT_6_R ligands with low basicity, the positive ionizable group, which was present in pharmacophore models developed in previous studies [22], is now not included in the model. The model is composed of three features: aromatic moiety (R7), hydrogen bond acceptor(A1) and hydrophobic moiety (H2). The features are arranged in the triangle-like shape with the distance between the aromatic ring and hydrogen bond acceptor equal to 2.77 Å, between hydrogen bond acceptor and hydrophobic feature: 2.43 Å, and 4.80 Å between the aromatic feature and hydrophobic moiety.

**Figure 3:**
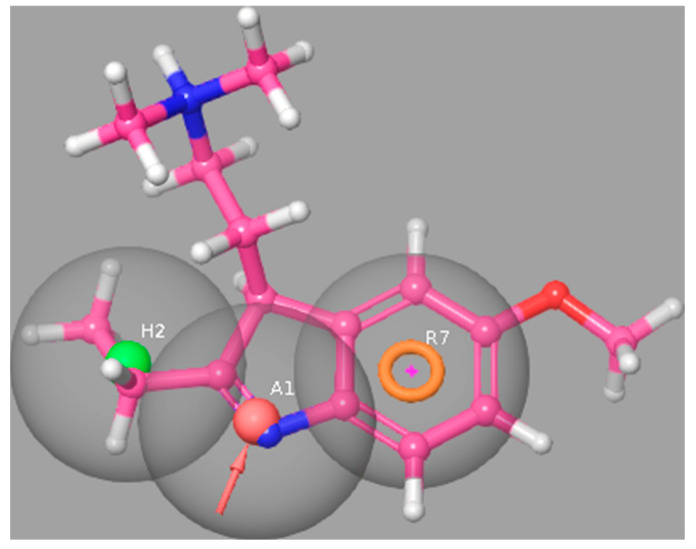
5-HT_6_R ligand (CHEMBL267615, *K_i_* = 16 nM) mapped to the pharmacophore model of the 5-HT_6_R ligands.

Mapping of the example 5-HT_6_R ligand (CHEMBL267615, *K_i_* = 13 nM) on this model is presented in Figure 3.

**Figure 4:**
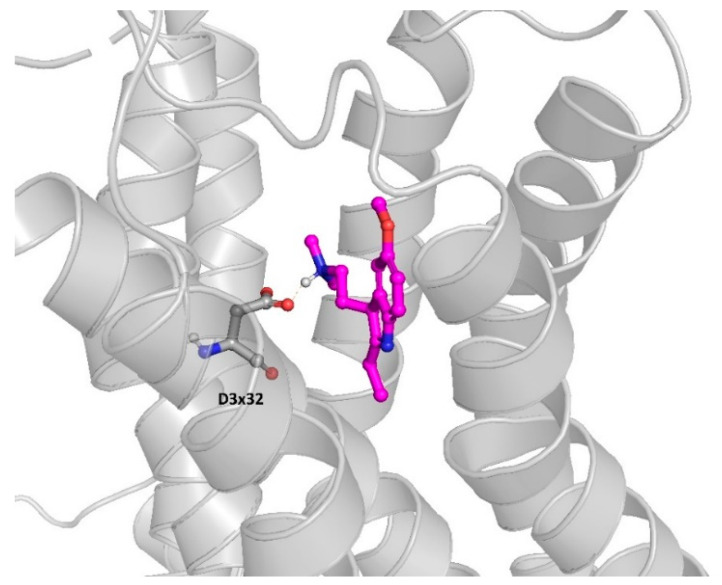
5-HT_6_R ligand (CHEMBL267615) docked to the 5-HT_6_R homology model.

In order to strongly support the compound activity via molecular modeling approaches, docking studies were carried out in the next step (inactive-state homology model of 5-HT_6_R deposited in the GPCRdb [50] database was used, and the docking was carried out in Glide [51] from the Schrödinger Suite 2022). The models were created based on the GPCRdb homology modeling pipeline [52]. It uses a chimeric approach in which a single template is selected as a main template; however, the template is screened locally and when a better template for a particular protein region is found, it is used to model the respective protein fragment The example ligand-receptor complex obtained for CHEMBL267615 is presented in Figure 4.

The compound presented in Figure 4 fits well in the 5-HT_6_R binding site, forming a number of hydrophobic and polar interactions. Most importantly, the charge-assisted hydrogen bond with the aspartic acid from the third transmembrane helix (D3x32 according to the GPCRdb numbering) is formed, but other important residues indicated as important to 5-HT_6_R activity also make contact with the compound, such as C3x36, S5x43, F6x51, F6x52, etc.

**Figure 5:**
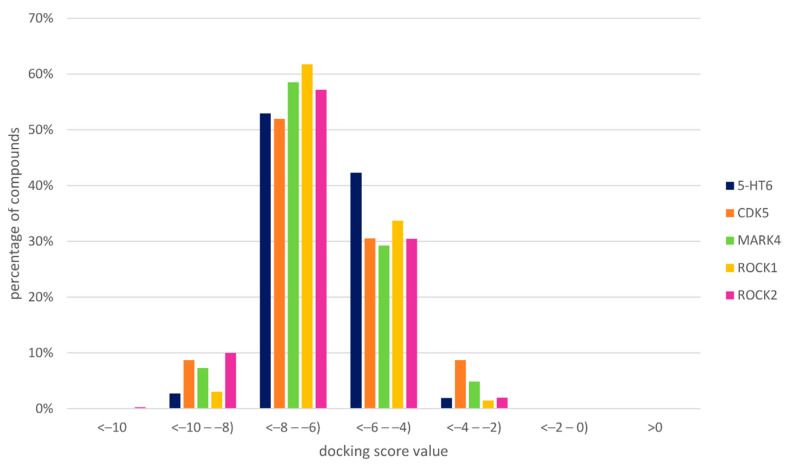
Distribution of docking score values to 5-HT_6_R homology model.

Additionally, the kinase inhibitors were docked to the 5-HT_6_R homology model in an analogous manner. The distribution of the docking score values for particular compound sets were examined (Figure 5, examples of docking poses are provided in the subsequent chapters).

The analysis of docking scores to 5-HT_6_R indicates that the distribution of their values is similar to both 5-HT_6_R ligands and ligands of the examined kinases. Although, the low docking score value does not guarantee the desired activity profile, its favorable value increases the probability of biding to the considered protein. For all ligand sets, the highest fraction of docking score values falls in the range of −8 to −6, and the second most populated group of values is between −6 and −4. CDK5, MARK4, ROCK I and ROCK II ligands possess a slightly higher fraction of compounds with a docking score between −4 and −2, but at the same time, for these targets, there is also a higher number of compounds with docking score values between −10 and −8 (in comparison to compounds active towards 5-HT_6_R).

In addition, the compounds were evaluated in terms of their ability to penetrate the blood–brain barrier. This was carried out via the determination of logP (calculations were performed in InstantJChem, https://chemaxon.com/products/instant-jchem [53]) for analogous compound sets, as in the case of docking. It was previously reported that logP values for the majority of drugs fall in the range of −0.5–6 [54]; however, the optimal logP range was set to 1.5–2.5 [55]. All the examined ligands fall in the similar logP distribution, with the majority of ligands adopting predicted logP values between 3 and 4 (Supplementary Materials Figure S1).

## 3. 5-HT_6_R/MARK4 as Dual Target Approach in Search for Therapeutic Solution against AD

Concerning the kinases, the mitogen-activated protein kinases (MAPKs) govern meaningful cellular programs and are crucial intermediate pathways in signaling, while microtubule affinity-regulating kinase 4 (MARK4) is a part of the kinases family recognized for actively phosphorylating neural microtubule-associated proteins (MAPs), i.e., MAP2, MAP4, and especially important for AD, tau protein. The kinase MARK4 is a member of the Ser/Thr kinase family and has been confirmed as a significant contributor in phosphorylating specific residues of tau, followed by its accumulation, and contributing in tauopathy. Phosphorylated tau also leads to neurofibrillary deposits and the formation of APP [56]. Tau phosphorylation effects are, therefore, correlated with neurodegeneration. Consequently, an overexpression of MARK4 is associated with numerous neurodegenerative disorders and neuropathy [57,58]. Thus, inhibiting MARK4 can be considered essential to cure some neurodegenerative diseases, including AD [59,60].

On the other hand, the highly important role of serotonin and 5-HT receptors in AD, particularly accented in the case of the 5-HT_6_R due to its unique function and CNS distributions, seems to be indisputable in light of the results of research conducted for over 20 years. Furthermore, recent lines of evidence, based on the fluorescence binding study, isothermal calorimetry, molecular docking and MD simulation studies for estimating the binding affinity and inhibiting potential of serotonin with MARK4, have demonstrated serotonin as an inhibitor of this important kinase. Hence, targeting MARK4 by serotonin opens a “new gate” in managing the clinical manifestations of neurodegenerative diseases such as AD and dementia [60].

In contrast to the 5-HTR-nonselective serotonin, selective 5-HT_6_R agents that also inhibit MARK4 would offer the possibility of a specific and better controlled pharmacological profile, thus guaranteeing more favorable therapeutic effects. In this context, the search for new structures of dual modulators of MARK4 and 5-HT_6_R gives new hope for a breakthrough in AD treatment, which is highly justified taking into account the signal transduction pathways at the cellular level. The question then arises as to whether it is possible to find these suitable double-agent structures from a chemical point of view.

Although lines of evidence indicate hundreds of 5-HT_6_R ligands with nanomolar affinities and different intrinsic profiles, the number of identified families of MARK4 inhibitors is much lower, and it is difficult to find any report on compounds simultaneously showing both MARK4 and 5-HT_6_R action in a high enough activity range. Among more than 100 dual MARK4/5-HT_6_R records in the CHEMBL database, none have been found to exhibit submicromolar effects for both purposes simultaneously. However, this state of the art does not seem to be associated with a distinct structural limitation but, more probably, with a lack of attention to this direction of research, until now.

**Figure 6:**
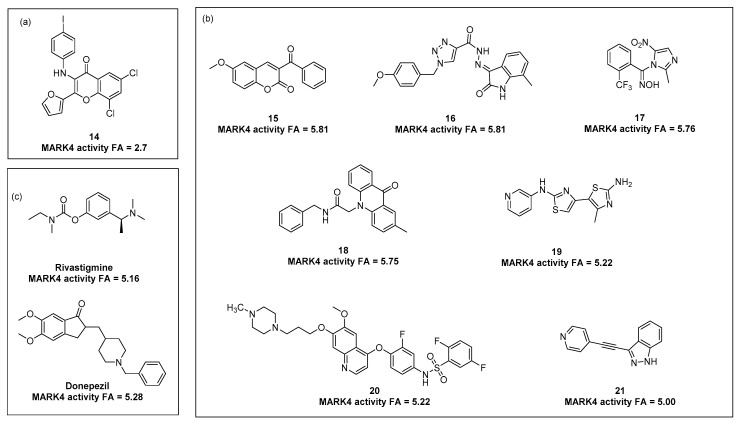
Representatives of weak and moderate MARK4 inhibitors with the activity descriptors; (**a**) the weak 3-N-aryl substituted-2-heteroarylchromoneinhibitor **14** [54] in comparison to the moderate 3-benzoylcoumarinMARK4 inhibitor; (**b**) the moderate inhibitors from various heterocyclic families **15 [55]**, **16** [56], **17** [57], **18 [58]**, **19 [59]**, **20 [60]**, **21** [61]; (**c**) MARK4 inhibitory properties of rivastigmine and donepezil [50]. To compare the activity expressed in various ways (IC50, *K*_d_ or %inhibition at a given concentration), the formal inhibition activity descriptor (FA) was used, i.e.,: FA = p*IC*_50_ (IC_50_); FA = p*K*_d_ (*K*_d_), FA = *p*(test concentration/0.02 × %inhibition).

Current lines of evidence show various chemical families of MARK4 inhibitors, estimated using different assays and activity descriptors, i.e., % inhibiting at concentrations of 1 µM or 10 µM, as well as IC_50_ or *K*_d_. The compounds can be classified into the following activity categories: weak (IC_50_ in millimolar range), moderate (1 µM < IC_50_ < 1 mM) and potent MARK4 inhibitors (IC_50_ < 1 µM). In order to compare the activity expressed in various ways (IC50, *K*_d_ or %inhibition at a given concentration), a formal inhibition activity descriptor (FA) was used (Figure 6, Table S1 in Supplementary Materials).

In 2018, Parveen et al. [61] described a series of novel 3-*N*-aryl substituted-2-heteroarylchromones with inhibitory properties toward MARK 4. Although the compounds displayed rather weak millimolar action, the results can be used for pharmacophore hypotheses in a wider structural consideration, and SAR analysis gave some valuable conclusions demonstrating the favorable role of moderate electro-withdrawing and lipophilic substituents (halogens) in both occurring aryl rings (**14**, Figure 6a).

A significant increase in activity was achieved through the replacement of the chromone core with a bioisosteric scaffold of 3-benzoylcoumarin substituted at the aromatic coumarin part. According to the studies described by Shen et al., the methoxy-substituent provides the most potent action with preferable position 6 compared to 8 (**15**, Figure 6b) to give inhibiting action at lower micromolar concentrations [62]. However, other lines of evidence indicate the wider chemical diversity of moderate MARK4 inhibitors with micromolar activities, including: derivatives of 1,2,3-triazole-4-carbohydrazide [63], imidazole oximes (**17**) [64], acridine derivatives (**18**) [65], pyridine derivative of dithiazole (**19**) [66], the extended structure of 2,5-difluoro-*N*-(3-fluoro-4-(6-methoxy-7-(3-(4-methylpiperazin-1-yl)propoxy)quinolin-4-yloxy)phenyl)benzenesulfonamide (**20**) [67] and the more condensed structure of 3-(2-(pyridin-4-yl)ethynyl)-1H-indazole (**21**) [68].

Interestingly, the studies of Shamsi et al. [57] demonstrated the moderate micromolar MARK4 inhibiting action for known AD drugs acting as AChE inhibitors, i.e., donepezil and rivastigmine; slightly more potent in the case of donepezil (Figure 6c).

**Figure 7:**
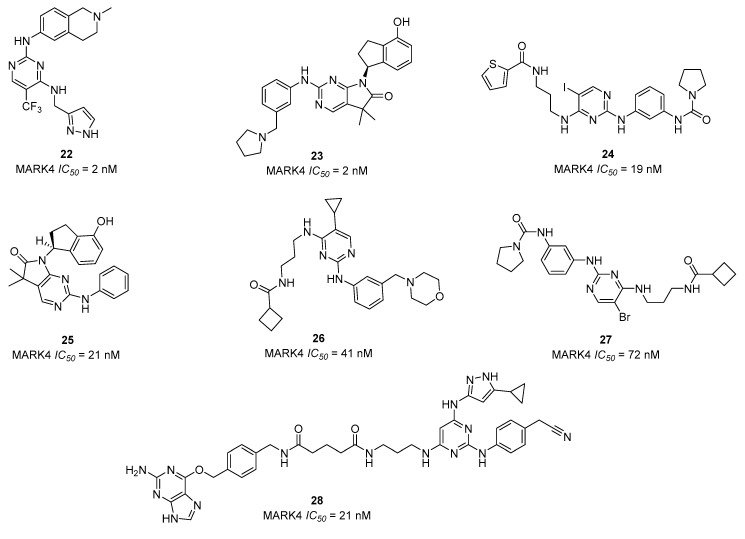
The most potent MARK4 inhibitors (IC_50_ < 100 nM) **22 [62]**, **23, 24, 26 [63]**, **25 [64]**, **27** [65], **28 [66]**.

The active MARK4 inhibitors, described with submicromolar to have a low-nanomolar range of action, contain the indispensable central core of the pyrimidine, which appears to be a required pharmacophore feature in interactions with this kinase, while a variety of the remaining substitutions enhances the inhibitory power [69,70,71,72,73,74]. The most active pyrimidine MARK4 inhibitors **22**–**28** (IC_50_ < 100 nM) are shown in Figure 7 [69,70,71,72,73].

**Figure 8:**
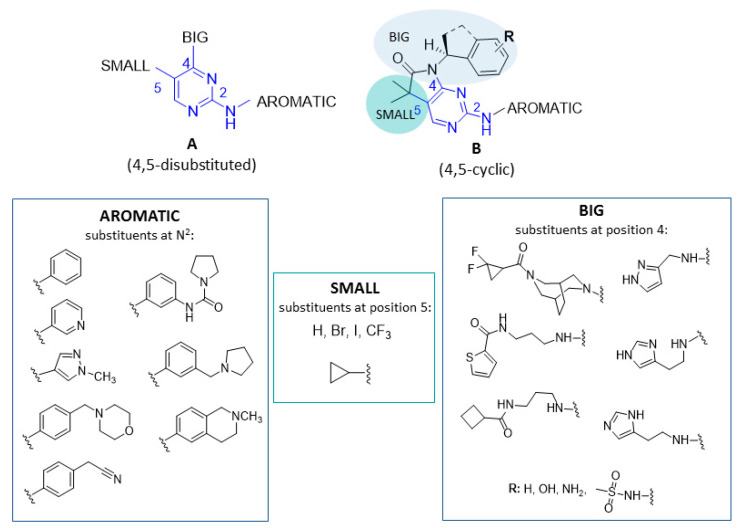
General structures (**A**,**B**) of the potent MARK4 inhibitors (IC_50_ < 1 µM) described [14,15,16,17,18,19]. The common feature of pyrimidine 2,4,5-trisubstituted (**A**) or 2-substituted-4,5-cyclic (**B**) in blue, the favorable substituents in rectangular frames.

A qualitative structure-activity relationship (SAR) analysis of the potent MARK4 inhibitors (IC_50_ < 1 µM) indicates two general structures based on pyrimidine core (Figure 8, see also Table S1 in Supplementary Materials).

Both groups (A and B) include different aromatic substituents attached to the amine group in position 2 of the pyrimidine ring with benzylpyrrolidine and 2-methyl-1,2,3,4-tetrahydroisoquinoline in the most potent compounds.

Other crucial features in these structures are substituents at positions 4 and 5 of the pyrimidine ring, depending on these substituents, structures can be divided into 4,5-disubstituted (A) and 4,5-cyclic (B). The substituent at position 5 is small and lipophilic, while substituents occurring at position 4 are rather bigger and contain an amide group and/or a heteroaromatic ring. In the case of 4,5-disubstituted pyrimidines, trifluoromethyl, iodine, bromine and cyclopropane seem to be the most favorable small substituents at position 5, while in position 4, the best results were obtained for the amine group linked by different alkyl chains with pyrazole, thiophene-2-carboxamide or cyclobutene carboxamide. For the 4,5-cyclic pyrimidines, the pyrimidine is condensed with 3,3-dimethylpyrrolidin-2-one, with another moiety attached by nitrogen in the pyrrolidine ring. Two methyl groups function as a small substituent at position 5 and the amide group with attached moiety (2,3-dihydroindene or ethylbenzene derivatives) serves as the bigger substituent at position 4. Additionally, an electron rich substituent (amine, methylsulfonamide or hydroxyl group) at the phenyl ring in the big substituent positively influenced the MARK4 inhibitory action.

However, lines of evidence indicate one unique compound **28** (Figure 7), which, unlike other discussed structures, possesses a very big substituent at position 6 instead of the small substituent at position 5; however, it still presents very good activity (IC_50_ = 21 nM).

**Figure 9:**
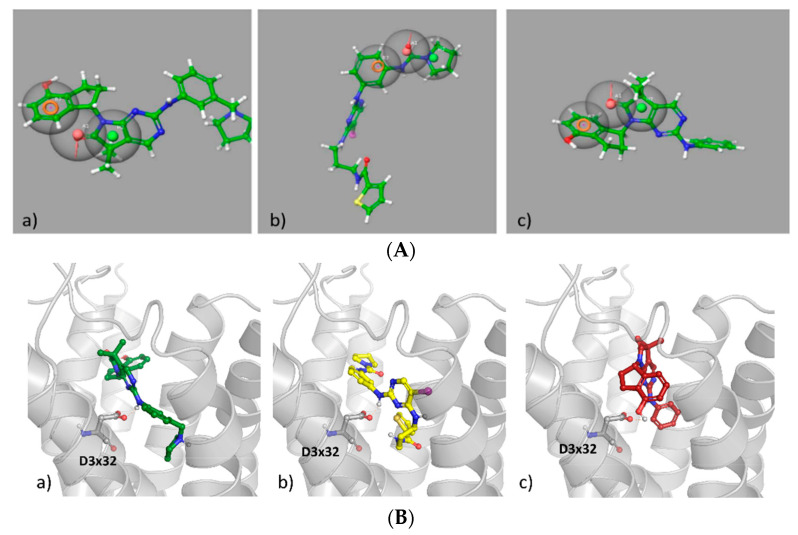
Examples of the most active MARK4 inhibitors: (**A**) mapped on the 5-HT_6_R pharmacophore model; (**B**) docked to the homology model of 5-HT_6_R; (**a**) **23,** (**b**) **24,** (**c**) **25**.

In order to examine possible dual MARK4/5-HT_6_R action, all potent MARK4 inhibitors **22**–**28** (Figure 7) were fitted to the pharmacophore model of 5-HT_6_R, and a docking study to the 5-HT_6_R homology model was carried out. The results for the most active inhibitors **23**, **24** and **25** are shown in Figure 9.

## 4. 5-HT_6_R/ROCKI/ROCKII as Multitarget Approach to AD Therapy

In 1995, Rho-associated coiled-coil-containing protein kinase, otherwise known as ROCK, was first identified and described as a major effector of RhoA [75]. This protein with a molecular mass of ~160 kDa belongs to the RhoA subfamily and the Ras GTPase superfamily with 25% homology to Ras [76]. Their structure comprises a N-terminally located catalytic Ser/Thr kinase domain, followed by a coiled-coil-forming region (~600 amino acids) with a Rho-binding domain (RBD) and a pleckstrin-homology (PH) domain with a cysteine-rich repeat at the C terminus [77]. Two mammalian isoforms of ROCK, ROCK I (ROCK-β, Rho-kinase β, or p160) encoded by a gene located on chromosome 18 and ROCK II (ROCK-α, p164) encoded by a gene located on chromosome 2, can be distinguished [78,79]. Despite a high structural similarity at approximately 65% overall amino acid identity and approximately 92% identity within the N-terminal kinase domain, these homologs have different locations in the body and, thus, different physiological functions have been identified for each [80].

In general, by phosphorylation of various molecular substrates, kinases ROCK are involved in many processes including cell contraction, adhesion, migration, growth, proliferation, inflammation, apoptosis and other various cellular functions [81]. Moreover, studies indicate that the activation of the RhoA/ROCK signaling pathway seems to induce Aβ aggregation [80], phosphorylated tau formation [82], neuroinflammation [83], synaptic damage [84], and other mechanisms, ultimately leading to AD [85].

Over the past decade, a whole host of structures have emerged as ROCK inhibitors for use in certain pathological conditions [86,87,88,89,90,91], including central nervous system diseases such as AD, Parkinson’s disease (PD) and Huntington’s disease (HD) [85,92,93]. So far, none have been sufficient for use in the treatment of neurodegenerative diseases. Furthermore, in the current literature, there are a lack of compounds with multitarget effects on ROCK and another dementia-related targets, such as 5-HT_6_R.

**Figure 10:**
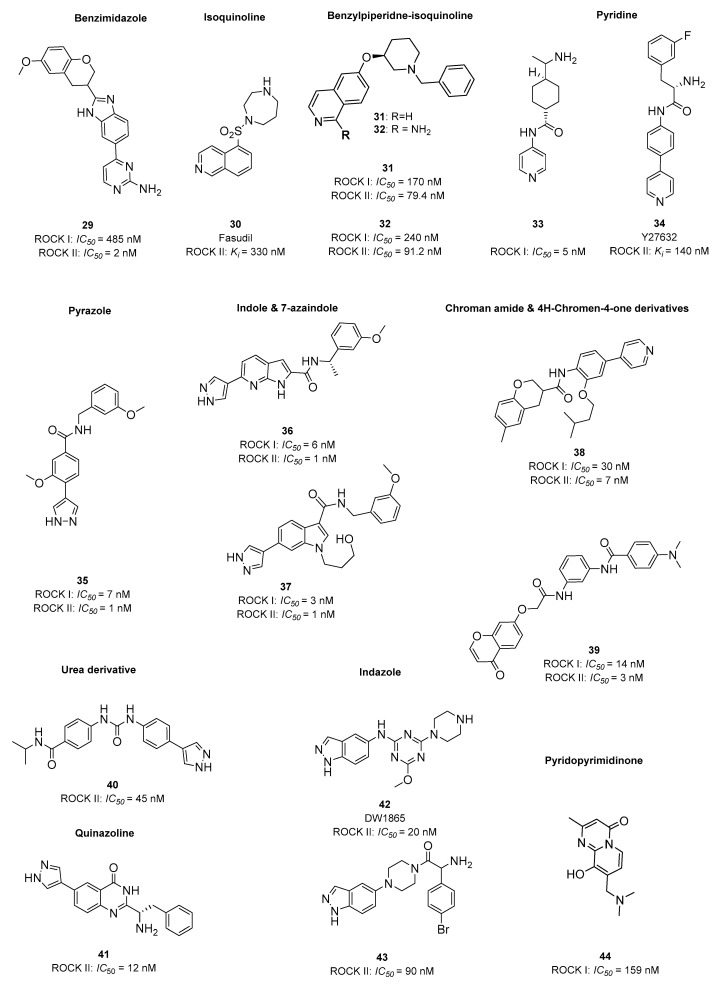
Examples of potent ROCK inhibitors from different chemical classes**: 29 [94], 31 [95]**, **32** **[95]**, **33 [96], 34 [97], 35 [98], 36 [99], 37 [100], 40 [102]**, **41** [103], **42** [106], **43** [108], **44** [CHEMBL1988581]. ROCK inhibiting potency expressed with either *IC*_50_ or *K_i_* values (nM).

Inhibition of ROCK I/II kinases has become a dynamically developing trend in recent years, as evidenced by the huge number of compounds from various chemical classes. Among them, it is possible to distinguish compounds belonging to the following chemical groups: benzimidazole **29 [94]**, isoquinoline **30**, bezylpiperidine-isoquinoline (**31, 32**) **[95]**, pyridine (**33, 34**) [96,97], pyrazole (**35**) [98], indole, azaindole (**36, 37**) [99,100] amide-chroman derivatives [101], urea derivatives(**40**) [102], quinazoline (**41**) [103], indazole (**42, 43**) [104,105,106] and pyridopyrimidinone (**44**) [CHEMBL1988581] (Figure 10), as well as benzothiazole, benzathiophene, aminofurazane, and boron derivatives [107].

One of the first and most important ROCK inhibitors was the isoquinoline derivative fasudil **30** [109], approved by the FDA for human use in 1995 in Japan for the treatment of cerebral vasospasm [110]. The compound is moderately potent with a *K_i_* of 330 nM and its structure consists of an isoquinoline ring, linked via a sulfonic group to the homopiperazine ring. Based on the current literature, it is worth noting that the sulfone moiety is also found in a great amount of potent 5-HT_6_ ligands.

To date, fasudil, as well as its analogues, are the most investigated ROCK inhibitors. Many studies have shown that the compound improves memory deficits, significantly reduces Aβ and p-tau protein levels, restores cognitive function, reduces oxidative stress, and decreases neuronal apoptosis in the hippocampus [111,112,113,114].

The compound Y-27632 **34** (Figure 10) and its analogues that have the aminopyridine core were synthesized by Yoshitomi Pharmaceuticals [115]. Moreover, compounds consisting of an aromatic ring directly attached at position 4 of the pyridine, azaindole, or pyrimidine already showed activity at the nanomolar level [97]. SAR analysis indicated that a large aromatic surface area hiding in the kinase active site and the additional presence of the NH moiety as hydrogen bond donors/acceptors significantly increases the inhibitory potency [116,117].

The indazole scaffold reported mainly by GlaxoSmithKline Pharmaceutical and Lee’s team has provided a number of compounds that can be considered potent inhibitors of ROCK I/II [104,105]. It should be noted that several essential 5-HT_6_ ligands, such as Cerlapirdine (**2**, Figure 1), developed by Pfizer, also contain a central core of the indazole [30]. In addition, many compounds in this chemical class have piperazine, 1,3,5-triazine [106] or 1,3-diazine (pyrimidine) moieties in their structure, which may also be a required pharmacophore feature of ROCK kinases. Importantly, these elements are a crucial structural feature of many 5-HT_6_ ligands, fitting into the current 5-HT_6_R pharmacophore.

**Figure 11:**
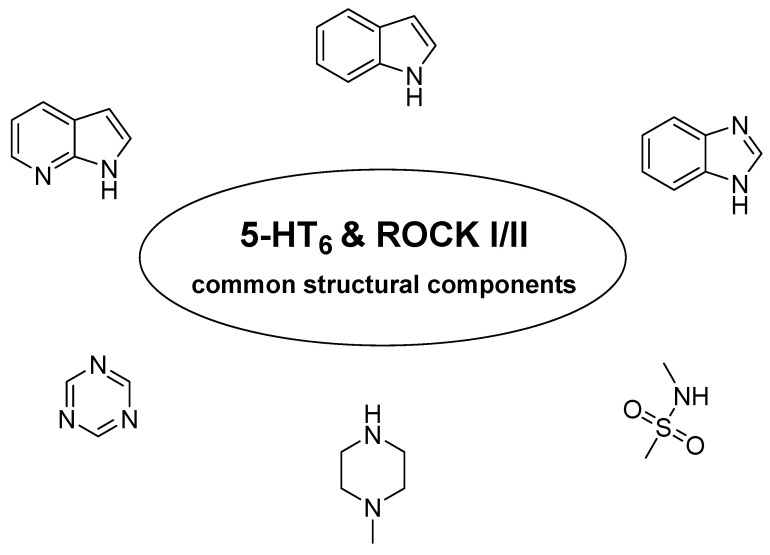
Common structural elements found in 5-HT_6_R ligands and ROCK I/II inhibitors.

The extensive literature used for this review also identified indole and 7-azaindole fused rings as structurally important moieties for both 5-HT_6_R ligands and ROCK I/II kinase inhibitors (Figure 11). All the structural similarities indicated above point to a real opportunity to create compounds with multitarget action on 5-HT_6_/ROCK I/II.

**Figure 12:**
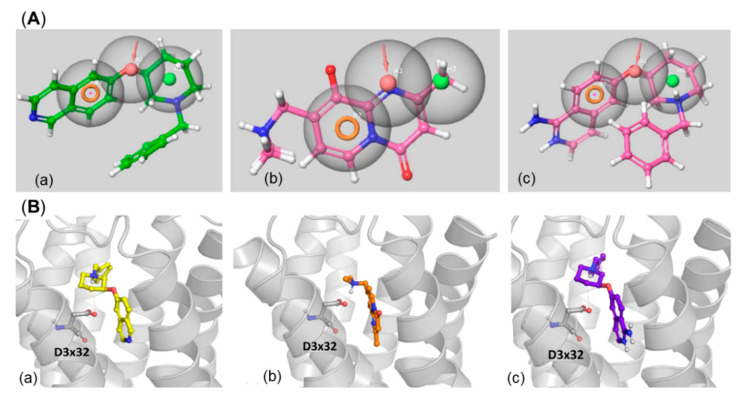
Examples of ROCK I/ROCK II ligands: (**A**) mapped to the 5-HT_6_R pharmacophore model; (**B**) docked to homology model of 5-HT_6_R; (**a**) **31**, (**b**) **44**, (**c**) **32**.

The potency of ROCK I and ROCK II inhibitors to also constitute good 5-HT_6_R ligands was tested in the following manner: all ROCK I and ROCK II data present in the ChEMBL database were filtered according to the activity values (*K_i_* or *IC*_50_), which were supposed to be lower than 500 nM to consider the compound as active. There were 983 such inhibitors of ROCK I, and 1841 compounds inhibiting ROCK II. The majority of those ligands (80% and 76%, respectively) were successfully mapped on the 5-HT_6_R pharmacophore model, with example mappings presented for **31**, **32** and **44** in Figure 12A.

Analogously to the MARK4 inhibitors, the ROCK I and ROCK II ligands were also docked to the 5-HT_6_R homology model (Figure 12B).

Despite the correct fitting of the ligands to the pharmacophore model, they also form energy-preferable complexes with the 5-HT_6_R. All the compounds presented in Figure 12B form a hydrogen bond with D3x32 (**31** and **32** via piperidine moiety, **44** via the amine part). **31**–5HT_6_R complex possesses an additional hydrogen bond interaction between the primary amine group and A5x43. All the compounds also interact with 5-HT_6_R via pi-pi stacking with F6x52 (**31** forms also pi-pi contact with F6x51).

## 5. 5-HT_6_R/CDK5 as Possible Dual Target Approach in Search for Innovative Therapy

First purified from bovine brain in 1992 [118], cyclin-dependent kinase 5 (CDK5) belongs to the family of proline-directed serine/threonine kinases and its gene is located on chromosome 7q36. The amino acids sequence of CDK5 is highly homologous to the sequence of other members of the CDKs family. In cells, it is responsible for various mechanisms including metabolic pathways, cell division and activation of transcriptional factors. To maintain its action, this kinase binds with unique activators such as p35 and p39 (expressed only in the CNS), the structure of which is more distinctive than typical CDKs. Structurally, the CDK5 protein consists of the N-terminus, C-terminus, ATP binding domain, activator binding domain, hinge region, PSSALRE helix and Tloop. Functions of the PSSALRE helix, Tloop, and ATP binding domain, which are essential for activation of CDK5, can be changed by different post-translational modifications (PTMs). Lines of evidence show that PTMs extend the functionality of the protein [119,120,121].

CDK5 in the human body can be found mainly in the central nervous system (CNS), where it participates in neuron migration, neurite overgrowth and synaptogenesis. Apart from CNS, CDK5 is also present in pancreatic β cells, corneal epithelial cells, and monocytes, where it is responsible for apoptosis, cell motility and cell cycle progression. Moreover, in previous years, CDK5 action was also proved to be associated with dopaminergic signaling, neurotransmitter release, and membrane cycling [122]. Concerning its mechanism of action, the aforementioned protein was suggested as a new therapeutic target for cancer [123,124,125], along with CNS diseases including AD, HD, stroke, and PD [126,127,128,129]. Increased activity of CDK5 is suggested as one of the causes of AD development. Dysregulation of this protein induces apoptosis of neuronal cells through various mechanisms, including Bcl-2, JNK3 and MEF2 [128].

**Figure 13:**
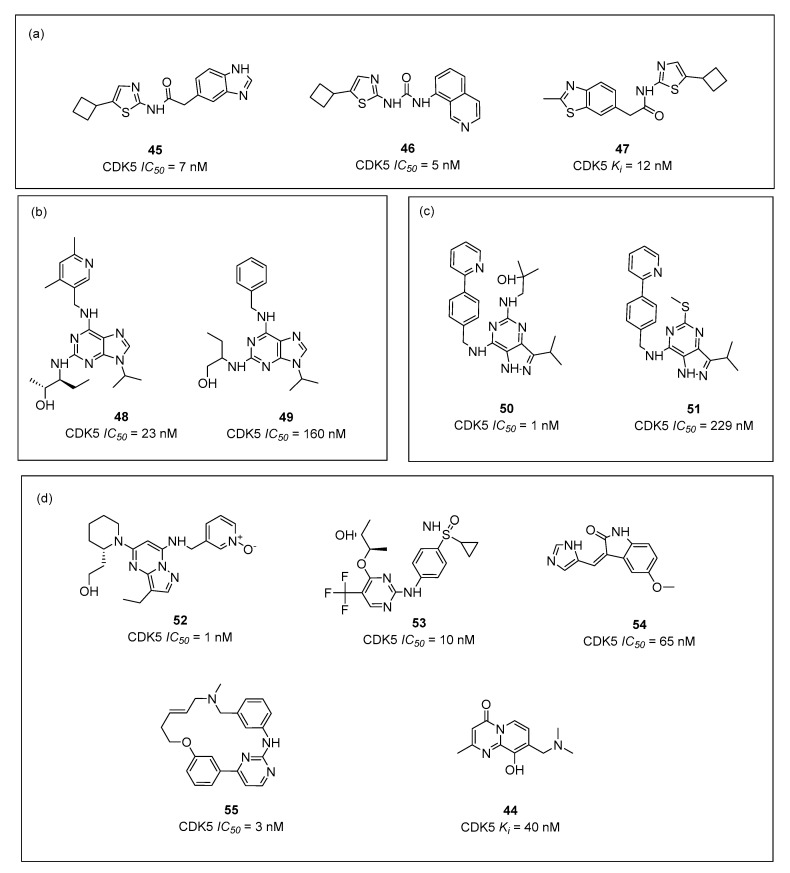
Examples of inhibitors of CDK5 with their affinities (*IC*_50_ or *K_i_*) within group (**a**–**d**) **45** [131], **46** [131], **47** [131], **48** [132], **49** [133], **50** [134], **51** [135], **52** [136], **53** [136], **54** [137], **55** [138], **44** (CHEMBL1988581).

Throughout the years, numerous different inhibitors with nanomolar affinity for CDK5 were discovered. Analyzing known inhibitors in the ChEMBL [130] database, three main chemical groups of the inhibitors (Figure 13) can be mentioned as follows: (i) cyclobutylthiazol-2-yl derivatives connected to either acetamide or urea (**45**, **46**, **47**, Figure 13a), (ii) 9-isopropyl-9H-purine derivatives (**48**, **49**, Figure 13b) and (iii) 3-isopropyl-1H-pyrazolo [4,3-d] pyrimidine (**50**, **51**, Figure 13c).

Along other inhibitors (Figure 13d), chemical structures vary genuinely, including structures of pyrazolo[1,5-a]pyrimidine (**52**), 2-aminopyrimidine (**53**), indoline-2-on (**54**), macrocycles (**55**) pyridopyrimidinone (**44**) and many others.

The first main group represents 5-cyclobutylthiazol-2-yl derivatives, from which SAR showed that the heteroaromatic ring connected in the 2-amino position and a small hydrophobic substituent in the thiazole 5-position increased the selectivity and potency towards the CDK5. Thiazole moiety can also be found in a few antagonists of 5-HT_6_ receptor, e.g., **12** with *K_i_* = 119 nM (Figure 1) [44].

Compounds containing 9-isopropyl-9H-purine scaffold (Figure 13b) having various hydroxyalkylamine substituents at the 5-position were shown to be the most potent within this group. As examples, highly potent reference kinase inhibitors: roscovitine, olomoucine and purvalanol A, may be mentioned. In a diverse compilation of 5-HT_6_ receptor antagonists, some of the structures (with nanomolar affinities towards 5-HT_6_R) can resemble the purine scaffold present in **48** and **49** (Figure 13b).

Structures within the third group (**50** and **51**, Figure 13c) include 3,5,7-trisubstituted pyrazolo[4,3-d]pyrimidine derivatives, in which several compounds showed very high kinase inhibiting potency (IC_50_ = 1 nM), introducing yet another scaffold in medicinal chemistry of CDKs inhibitors.

**Figure 14:**
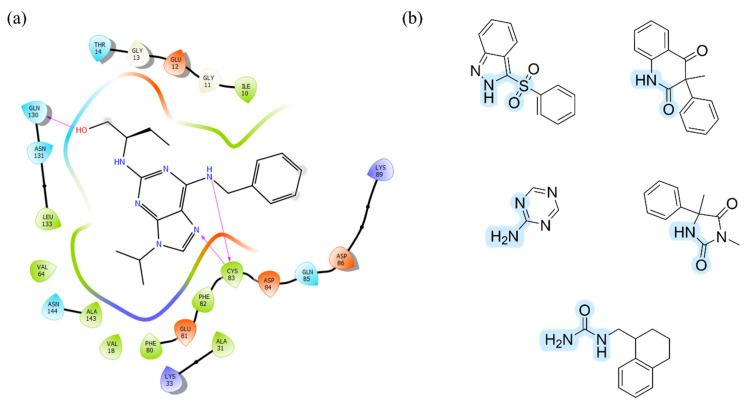
(**a**) 2D protein-ligand interactions for roscovitine—strong CDK5 inhibitor (generated using Schrodinger Suite); (**b**) The examples of structural fragments of 5-HT_6_R ligands favorable for interactions with Cys83 in ATP binding pocket of CDK5.

Importantly, several studies including X-ray and molecular modeling have indicated the pivotal role of cysteine (Cys83) in ligands binding to CDK5 in the ATP binding pocket [139,140]. This amino acid may be S-nitrosylated and, fascinatingly, the perturbation of such a process leads to the enhancement of dendrite development in cultured hippocampal neurons, which, of course, influences overall neuronal development [120]. Cys83, thanks to its characteristic structure, acts simultaneously as a hydrogen bond acceptor and donor. Hence, potent CDK5 inhibitors very often possess structural fragments that also consist of pairs, such as hydrogen bond acceptor and donor, placed closed to each other (Figure 14). Thus, it is possible to form two hydrogen bonds with the protein via interaction with Cys83 (Figure 14a) [141]. Interestingly, such chemical groups also occur in the structures of many 5-HT_6_R ligands, increasing the probability of their strong binding to CDK5 (Figure 14b).

Dozens of CDK5 inhibitors have reached clinical trials, mainly as therapeutics for various cancers. Dinaciclib (**52**, Figure 13d), for example, is currently undergoing phase 1 of the clinical trials for the treatment of breast cancer [142]. The most potent inhibitors reach the 1 nM affinity. Despite the huge number of highly active CDK5 agents, dual 5-HT_6_R/CDK5 continue to be an underexplored area of scientific research.

**Figure 15:**
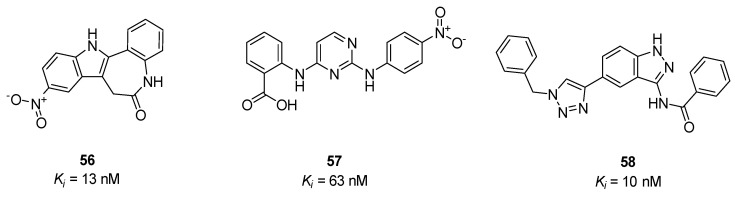
Examples of CDK5 ligands which were not successfully mapped to the 5-HT_6_R pharmacophore model.

In terms of exploring the structural possibility for the desirable CDK5/5-HT_6_R dual action, the CDK5 ligands, which were filtered according to the same criteria as ROCK I and ROCK II compounds, formed the set of 263 compounds. Among them, 219 compounds were properly mapped to the 5-HT_6_R pharmacophore model, indicating their high potency for possessing a 5-HT_6_R activity component. Examples of three (**56–58**) out of 44 compounds, which were not mapped to the model, are presented in Figure 15.

**Figure 16:**
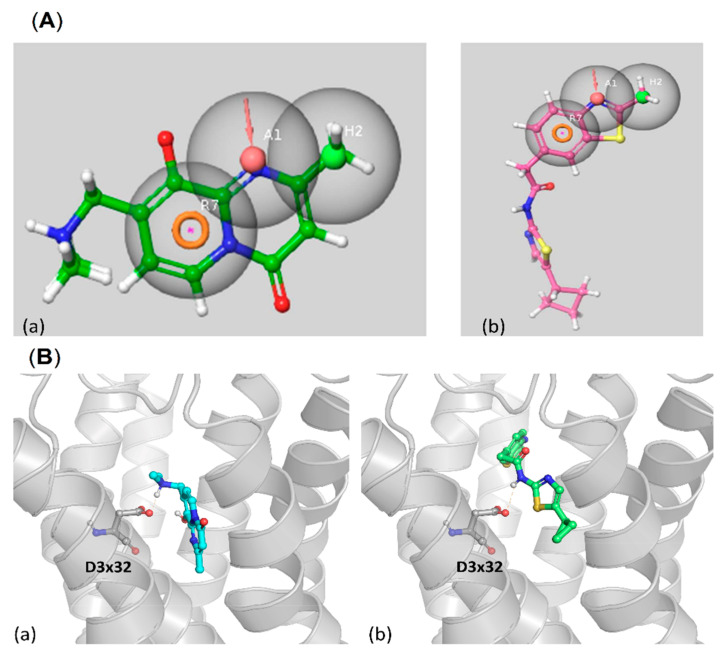
Examples of CDK5 ligands which were successfully mapped to the 5-HT_6_R pharmacophore model (**A**) together with their docking results (**B**); (**a**) **44**, (**b**) **47**.

In addition, the compounds were docked to the 5-HT_6_R homology model. Results for representatives (**44** and **47**) of both pharmacophore mapping and docking are presented in Figure 16. 

Both ligands (**44** and **47**) are well aligned to the pharmacophore features of 5-HT_6_R ligands (Figure 16A). Compound **44**, which has a smaller structure, is almost fully covered by the 5-HT_6_R pharmacophore model, in contrast to **47**, for which quite a significant part of the molecule is outside of the model. Despite this extending part, **47** is very well fitted to the three considered features. Compound **47** also did not enter very deeply into the 5-HT_6_R binding site, rather it occupies its upper part (Figure 16B). Nevertheless, both compounds are strongly fitted in their ligand-protein complexes through the extended network of polar and hydrophobic contacts. Additionally, both **44** and **47** form hydrogen a bond with D3 × 32 and pi-pi interaction with a phenylalanine cluster from the 6^th^ transmembrane helix of 5-HT_6_R.

## 6. Conclusions

As polypharmacology approaches may result in long-awaited breakthroughs in AD treatment, a computer-aided and visual analysis of the possibility of designing molecules with as yet unreported action via both 5-HT_6_R and the AD pathology-related kinase (MARK4, ROCKI/II or CDK5) within this paper has been performed. The deep insight into recent lines of evidence allowed us to identify the structural fragments that occur simultaneously in 5-HT_6_R agents and inhibitors of the considered above-mentioned kinases, as well as their appropriate bioisosteres. Interestingly, mapping the known kinase inhibitors to the 5-HT_6_R pharmacophore model showed the high potency of the majority of them to interact with the 5-HT_6_R. Hence, all potent pyrimidine-derived MARK4 inhibitors meet the 5-HT_6_R pharmacophore features criteria, as well as 80% of ROCKI, 76% of ROCKII and 83% of CDK5 ligands. Additionally, the results of docking to the 5-HT_6_R homology model confirmed the high probability of the investigated structures to form key interactions with this protein target. More than 60% of all four groups of the tested agents were docked with very good docking scores (values in the range between −10 and −6).

Summarizing, the overall analysis of the results from the pharmacophore-based and the docking-based (docking score values distribution) approaches indicated the high potency of inhibitors for all four investigated kinases to be also 5-HT_6_R antagonists. Furthermore, this very initial prediction of ADMET properties [143] for the most active kinase inhibitors that also fit in the 5-HT_6_R ligand pharmacophore features (**23**, **24**, **25**, **31**, **32**, **44** and **47**) demonstrated rather a satisfactory profile for most of them, comparable to that of the reference drug, donepezil (see Table S2, Figure S1 in Supplementary Materials).

Such results give real hope for the design of structurally novel anti-AD agents with pioneering multifunctional (dual) action. Simultaneously, it seems to be strongly justified to test already reported 5-HT_6_R ligands in terms of potency to inhibit the investigated herein kinases, as well as to examine ADMET properties for the most promising dual-target agents found following this process.

## Data Availability

Not applicable.

## Supplementary Materials

The supporting information can be downloaded at: https://www.mdpi.com/article/10.3390/ijms23158768/s1. References [61,62,63,64,65,66,67,68,69,70,71,72,73,143] are cited in the supplementary materials.
