# Supplementary material for: Multitargeting the Action of 5-HT6 Serotonin Receptor Ligands by Additional Modulation of Kinases in the Search for a New Therapy for Alzheimer’s Disease: Can It Work from a Molecular Point of View?
Source: Int J Mol Sci. 2022 Aug 7;23(15):8768. doi: 10.3390/ijms23158768 (PMC9368844; doi:10.3390/ijms23158768)
Supplement: Supplementary file 1 [file ijms-23-08768-s001.zip › ijms-1850675-supplementary.pdf]

Supplementary

Multitargeting the Action of 5-HT$_6$ Serotonin Receptor Ligands by Additional Modulation of Kinases in the Search for a New Therapy for Alzheimer's Disease: Can It Work from a Molecular Point of View?

Kinga Czarnota-Łydka, Katarzyna Kucwaj-Brysz, Patryk Pyka, Wawrzyniec Haberek, Sabina Podlewska* and Jadwiga Handzlik*

Table S1. MARK4 inhibitors described in literature

Cpd	Structure	SMILES	MARK4 inhibition				FA
			IC$_{50}$ (μM)	% (1 μM)	% (10 μM)	Kd (μM)	
**S1**	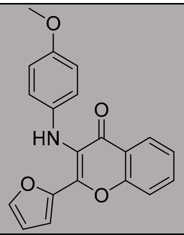	O=C1C(NC2=CC=C(OC)C=C2)=C(C3=CC=CO3)OC4=C1C=CC=C4	5560				2.26
**S2**	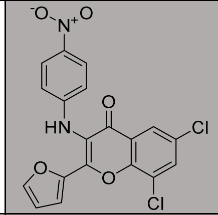	O=C1C(NC2=CC=C([N+]([O-])=O)C=C2)=C(C3=CC=CO3)OC4=C1C=C(Cl)C=C4Cl	2120				2.67
**S3**	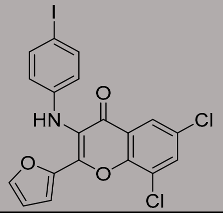	O=C1C(NC2=CC=C(I)C=C2)=C(C3=CC=CO3)OC4=C1C=C(Cl)C=C4Cl	1980				2.7

**S4**	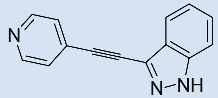	C1(C#CC3=CC=NC=C3)=NNC2=C1C=CC=C2			>50		**5**
**S5**	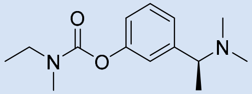	CCN(C(OC1=CC=CC([C@@H](N(C)C)C)=C1)=O)C	6.94				**5.16**
**S6**	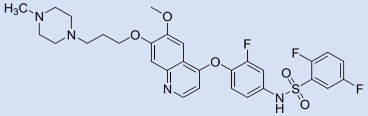	O=S(C1=CC(F)=CC=C1F)(NC2=CC=C(OC4=C3C=C(OC)C(OCCCN5CCN(C)CC5)=CC3=NC=C4)C(F)=C2)=O				5.975	**5.22**
**S7**	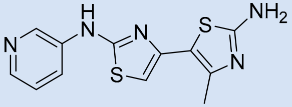	CC(N=C(N)S1)=C1C2=CSC(NC3=CN=CC=C3)=N2			83		5.22
**S8**	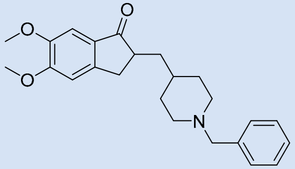	O=C1C(CC2=CC(OC)=C(OC)C=C21)CC3CCN(CC3)CC4=CC=CC=C4	5.3				**5.28**
**S9**	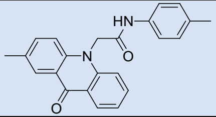	O=C(NC2=CC=C(C)C=C2)CN(C4=C3C=CC=C4)C1=CC=C(C)C=C1C3=O	4.5				**5.35**
**S10**	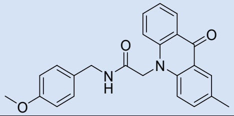	O=C(NCC2=CC=C(OC)C=C2)CN(C4=C3C=CC=C4)C1=CC=C(C)C=C1C3=O	2.20				**5.66**
**S11**	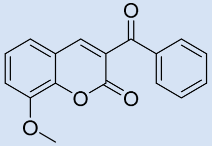	O=C1OC2=C(OC)C=CC=C2C=C1C(C3=CC=CC=C3)=O	2.09				**5.68**
**S12**	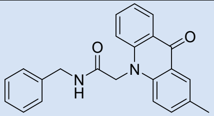	O=C(NCC2=CC=CC=C2)CN(C4=C3C=CC=C4)C1=CC=C(C)C=C1C3=O	1.8				**5.75**

**S13**	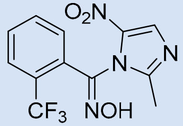	CC1=NC=C([N+]([O-])=O)N1C(C2=C(C(F)(F)F)C=CC=C2)=NO	1.74				**5.76**
**S14**	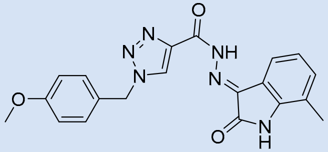	O=C(C1=CN(CC2=CC=C(OC)C=C2)N=N1)N/N=C3C(NC4=C/3C=CC=C4C)=O	1.54				5.81
**S15**	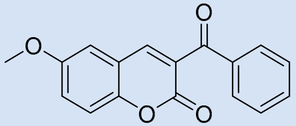	O=C1OC2=CC=C(OC)C=C2C=C1C(C3=CC=CC=C3)=O	1.3				**5.89**
**S16**	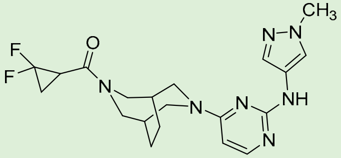	CN(N=C1)C=C1NC2=NC=CC(N3CC(CN(C(C4C(F)(F)C4)=O)C5)CCC5C3)=N2		59%			6.07
**S17**	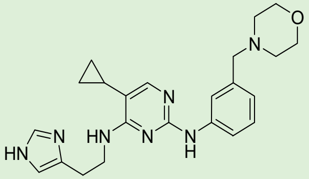	C1(NC2=CC=CC(CN4CCOCC4)=C2)=NC=C(C5CC5)C(NCCC3=CNC=N3)=N1	0.808				6.09
**S18**	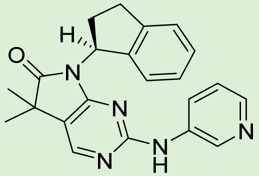	O=C(C1(C)C)N([C@]2([H])C(C=CC=C3)=C3CC2)C4=C1C=NC(NC5=CN=CC=C5)=N4	0.520				6.28
**S19**	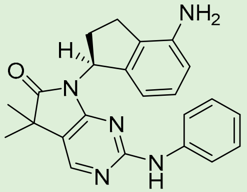	O=C(C1(C)C)N([C@]2([H])C(C=CC=C3N)=C3CC2)C4=C1C=NC(NC5=CC=CC=C5)=N4	0.450				6.35

**S20**	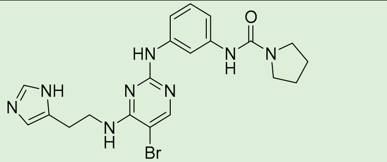	BrC1=CN=C(NC2=CC(NC(N3CCCC3)=O)=CC=C2)N=C1NCCC4=CN=CN4	0.338				6.47
**S21**	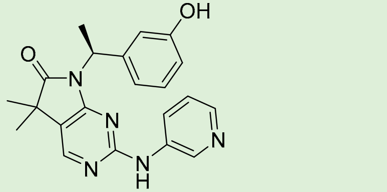	O=C(C1(C)C)N([C@@H](C)C2=CC=CC(O)=C2)C3=C1C=NC(NC4=CN=CC=C4)=N3	0.270				6.56
**S22**	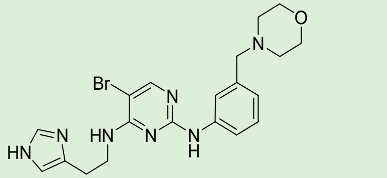	BrC1=CN=C(NC3=CC=CC(CN4CCOCC4)=C3)N=C1NCCC2=CNC=N2	0.214				**6.69**
**S23**	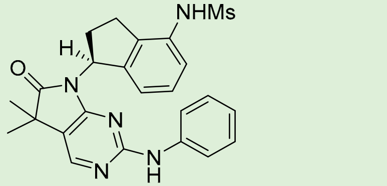	O=C(C1(C)C)N([C@]2([H])C(C=CC=C3NS(C)(=O)=O)=C3CC2)C4=C1C=NC(NC5=CC=CC=C5)=N4	0.180				6.75
**S24**	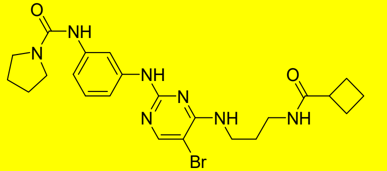	O=C(N2CCCC2)NC1=CC=CC(NC3=NC=C(Br)C(NCCCNC(C4CCC4)=O)=N3)=C1	0.072				**7.14**
**S25**	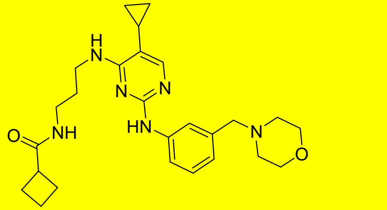	O=C(C3CCC3)NCCCNC1=NC(NC4=CC=CC(CN5CCOCC5)=C4)=NC=C1C2CC2	0.041				**7.39**

**S26**	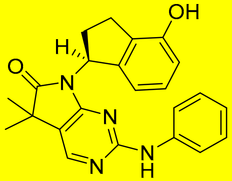	O=C(C1(C)C)N([C@]2([H])C(C=CC=C3O)=C3CC2)C4=C1C=NC(NC5=CC=CC=C5)=N4	0.021				7.68
**S27**	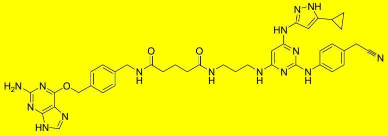	O=C(CCCC(NCC5=CC=C(COC6=NC(N)=NC7=C6N=CN7)C=C5)=O)NCCCNC1=CC(NC2=NNC(C4CC4)=C2)=NC(NC3=CC=C(CC#N)C=C3)=N1	0.021				**7.68**
**S28**	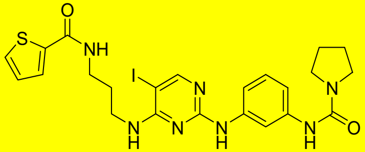	O=C(N2CCCC2)NC1=CC=CC(NC3=NC=C(I)C(NCCCNC(C4=CC=CS4)=O)=N3)=C1	0.019				**7.72**
**S29**	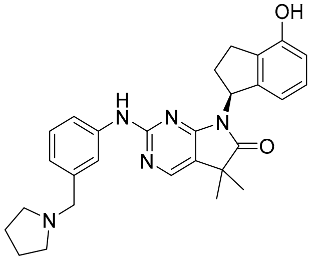	CC(C(N1[C@H]2CCC3=C2C=CC=C3O)=O)(C)C(C1=N4)=CN=C4NC5=CC=CC(CN6CCCC6)=C5	0.002				**8.70**
**S30**	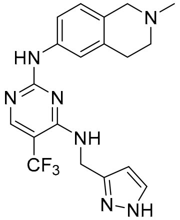	CN1CCC2=CC(NC3=NC=C(C(F)(F)F)C(NCC4=NNC=C4)=N3)=CC=C2C1	0.00201				**8.7**

The range of activity (FA range) is discriminated with different colors.

Formal activity (FA) descriptor of the inhibiting action, calculated according to the data provided [1-13], as follows:

a) FA = pIC_{50}
b) FA= pKd
c) FA = p(the given concentration/0.02 x %inhibition)

Table S2. Selected ADMET properties calculated with pkCSM software (http://biosig.unimelb.edu.au/pkcsm/prediction) [14] for the most active kinase inhibitors fitted in pharmacophore features of the 5-HT_6R ligands

		Absrorption		Distribution	Toxicity	
**Cpd**	**Structure**	**[1]Caco-2 permeability**	**[2]Intestinal absorption [%]**	**[3]CNS Permeability**	**hERG I inhibitor**	**[4]Oral Rat Acute Toxicity (LD_{50})**
**23**	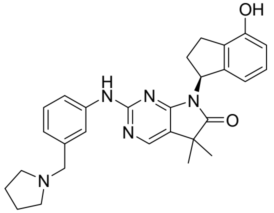	0.848	88.83	-2.11	No	3.207
**24**	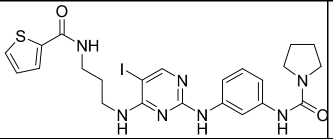	0.592	75.89	-2.553	No	2.344
**25**	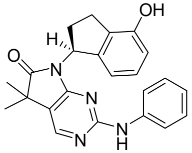	0.685	92.87	-1.933	No	2.628
**31**	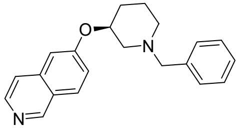	1.66	94.376	-1.257	No	3.069
**32**	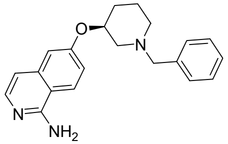	1.184	90.671	-1.908	No	2.802
**44**	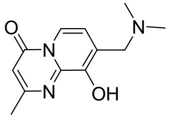	1.115	100	-3,317	No	2.623

**47**	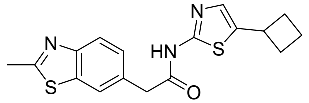	1.388	89.803	-1,886	No	2.974
**Ref.**	**Donepezil**	1.001	93.076	-1.933	No	2.886

[1]Caco-2 > 0.9 for high permeable compounds; [2]poor intestinal absorption < 30%; [3]CNS permeability: logPS> -2 (high), -3≤ logPS ≤-2 (moderate), logPS< -3 unable; [4]LD_{50} [mol/kg b.w.]. [5]Ref.

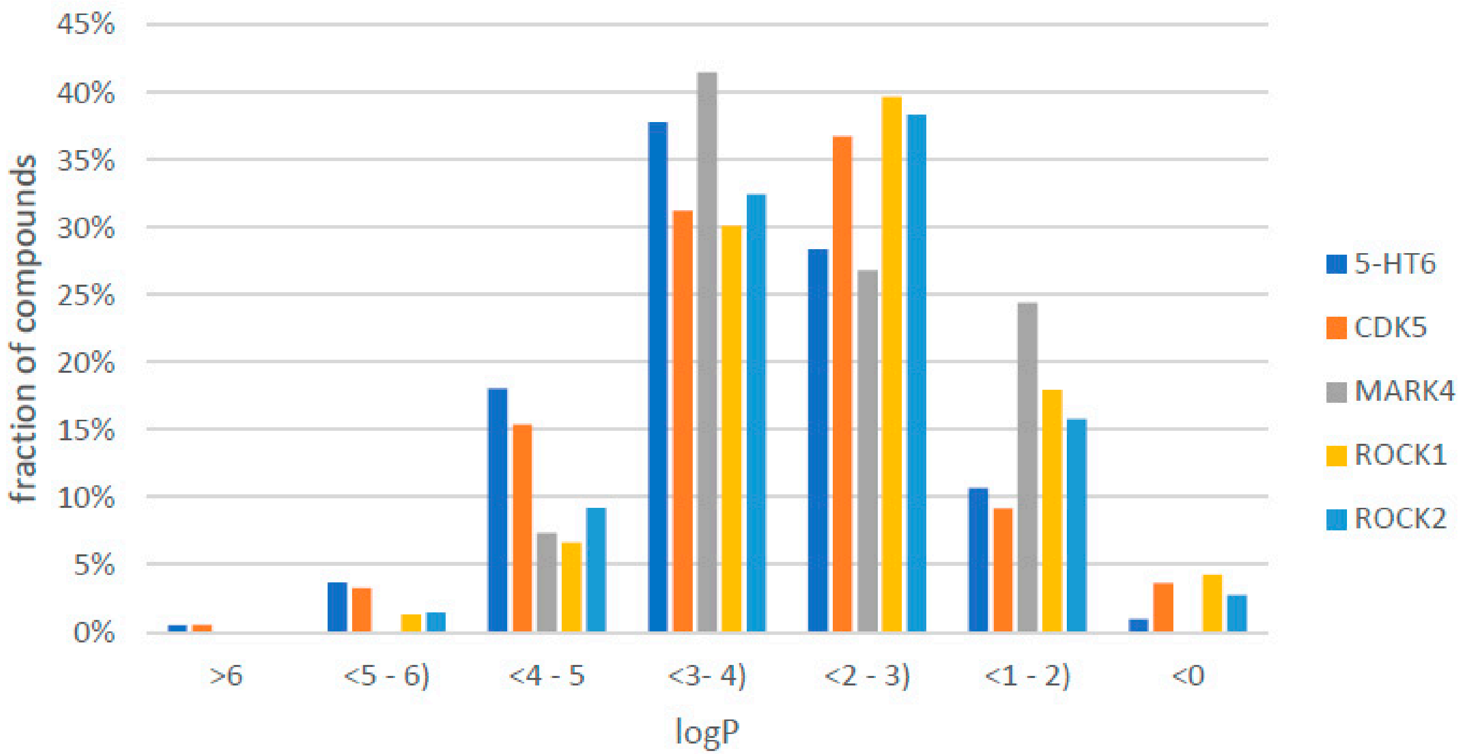

Figure S1. Distribution of logP values for the examined ligands.

.

References

1. Parveen, I.; Khan, P.; Ali, S.; Hassan, Md.I.; Ahmed, N. Synthesis, Molecular Docking and Inhibition Studies of Novel 3-N-Aryl Substituted-2-Heteroarylchromones Targeting Microtubule Affinity Regulating Kinase 4 Inhibitors. *European Journal of Medicinal Chemistry* **2018**, *159*, 166–177, doi:10.1016/j.ejmech.2018.09.030.

2. Shen, X.; Liu, X.; Wan, S.; Fan, X.; He, H.; Wei, R.; Pu, W.; Peng, Y.; Wang, C. Discovery of Coumarin as Microtubule Affinity-Regulating Kinase 4 Inhibitor That Sensitize Hepatocellular Carcinoma to Paclitaxel. *Frontiers in Chemistry* **2019**, *7*, doi:10.3389/fchem.2019.00366.

3. Aneja, B.; Khan, N.S.; Khan, P.; Queen, A.; Hussain, A.; Rehman, Md.T.; Alajmi, M.F.; El-Seedi, H.R.; Ali, S.; Hassan, Md.I.; et al. Design and Development of Isatin-Triazole Hydrazones as Potential Inhibitors of Microtubule Affinity-Regulating Kinase 4 for the Therapeutic Management of Cell Proliferation and Metastasis. *European Journal of Medicinal Chemistry* **2019**, *163*, 840–852, doi:10.1016/j.ejmech.2018.12.026.

4. Peerzada, M.N.; Khan, P.; Khan, N.S.; Avecilla, F.; Siddiqui, S.M.; Hassan, Md.I.; Azam, A. Design and Development of Small-Molecule Arylaldoxime/5-Nitroimidazole Hybrids as Potent Inhibitors of MARK4: A Promising Approach for Target-Based Cancer Therapy. *ACS Omega* **2020**, *5*, 22759–22771, doi:10.1021/acsomega.0c01703.

5. Voura, M.; Khan, P.; Thysiadis, S.; Katsamakas, S.; Queen, A.; Hasan, G.M.; Ali, S.; Sarli, V.; Hassan, Md.I. Probing the Inhibition of Microtubule Affinity Regulating Kinase 4 by N-Substituted Acridones. *Scientific Reports* **2019**, *9*, 1676, doi:10.1038/s41598-018-38217-8.

6. Ngoei, K.R.W.; Ng, D.C.H.; Gooley, P.R.; Fairlie, D.P.; Stoermer, M.J.; Bogoyevitch, M.A. Identification and Characterization of Bi-Thiazole-2,2′-Diamines as Kinase Inhibitory Scaffolds. *Biochimica et Biophysica Acta (BBA) - Proteins and Proteomics* **2013**, *1834*, 1077–1088, doi:10.1016/j.bbapap.2013.02.001.

7. Szabadkai, I.; Torka, R.; Garamvölgyi, R.; Baska, F.; Gyulavári, P.; Boros, S.; Illyés, E.; Choidas, A.; Ullrich, A.; Őrfi, L. Discovery of *N* -[4-(Quinolin-4-Yloxy)Phenyl]Benzenesulfonamides as Novel AXL Kinase Inhibitors. *Journal of Medicinal Chemistry* **2018**, *61*, 6277–6292, doi:10.1021/acs.jmedchem.8b00672.

8. Barile, E.; De, S.K.; Carlson, C.B.; Chen, V.; Knutzen, C.; Riel-Mehan, M.; Yang, L.; Dahl, R.; Chiang, G.; Pellecchia, M. Design, Synthesis, and Structure−Activity Relationships of 3-Ethynyl-1 *H* -Indazoles as Inhibitors of the Phosphatidylinositol 3-Kinase Signaling Pathway. *Journal of Medicinal Chemistry* **2010**, *53*, 8368–8375, doi:10.1021/jm100825h.

9. Li, F.; Liu, Z.; Sun, H.; Li, C.; Wang, W.; Ye, L.; Yan, C.; Tian, J.; Wang, H. PCC0208017, a Novel Small-Molecule Inhibitor of MARK3/MARK4, Suppresses Glioma Progression in Vitro and in Vivo. *Acta Pharmaceutica Sinica B* **2020**, *10*, 289–300, doi:10.1016/j.apsb.2019.09.004.

10. Faisal, M.; Kim, J.H.; Yoo, K.H.; Roh, E.J.; Hong, S.S.; Lee, S.H. Development and Therapeutic Potential of NUAKs Inhibitors. *Journal of Medicinal Chemistry* **2021**, *64*, 2–25, doi:10.1021/acs.jmedchem.0c00533.

11. Katz, J.D.; Haidle, A.; Childers, K.K.; Zabierek, A.A.; Jewell, J.P.; Hou, Y.; Altman, M.D.; Szewczak, A.; Chen, D.; Harsch, A.; et al. Structure Guided Design of a Series of Selective Pyrrolopyrimidinone MARK Inhibitors. *Bioorganic & Medicinal Chemistry Letters* **2017**, *27*, 114–120, doi:10.1016/j.bmcl.2016.08.068.

12. Drewry, D.H.; Annor-Gyamfi, J.K.; Wells, C.I.; Pickett, J.E.; Dederer, V.; Preuss, F.; Mathea, S.; Axtman, A.D. Identification of Pyrimidine-Based Lead Compounds for Understudied Kinases Implicated in Driving Neurodegeneration. *Journal of Medicinal Chemistry* **2022**, *65*, 1313–1328, doi:10.1021/acs.jmedchem.1c00440.

13. Gower, C.M.; Thomas, J.R.; Harrington, E.; Murphy, J.; Chang, M.E.K.; Cornella-Taracido, I.; Jain, R.K.; Schirle, M.; Maly, D.J. Conversion of a Single Polypharmacological Agent into Selective Bivalent Inhibitors of Intracellular Kinase Activity. *ACS Chemical Biology* **2016**, *11*, 121–131, doi:10.1021/acschembio.5b00847.

14. Pires, D.; Blundell, T.; Ascher, D. pkCSM: Predicting Small-Molecule Pharmacokinetic and Toxicity Properties Using Graph-Based Signatures. . *Journal of Medicinal Chemistry* **2015**, 58, 4066–72, doi: 10.1021/acs.jmedchem.5b00104
